# Nicotine Attenuates Activation of Tissue Resident Macrophages in the Mouse Stomach through the β2 Nicotinic Acetylcholine Receptor

**DOI:** 10.1371/journal.pone.0079264

**Published:** 2013-11-01

**Authors:** Andrea Nemethova, Klaus Michel, Pedro J. Gomez-Pinilla, Guy E. Boeckxstaens, Michael Schemann

**Affiliations:** 1 Department of Clinical and Experimental Medicine, Translational Research Center for Gastrointestinal Disorders (TARGID), University of Leuven, Leuven, Belgium; 2 Human Biology, Technische Universität München, Freising, Germany; University of California, Los Angeles, United States of America

## Abstract

**Background:**

The cholinergic anti-inflammatory pathway is an endogenous mechanism by which the autonomic nervous system attenuates macrophage activation via nicotinic acetylcholine receptors (nAChR). This concept has however not been demonstrated at a cellular level in intact tissue. To this end, we have studied the effect of nicotine on the activation of resident macrophages in a mouse stomach preparation by means of calcium imaging.

**Methods:**

Calcium transients ([Ca^2+^]_i_) in resident macrophages were recorded in a mouse stomach preparation containing myenteric plexus and muscle layers by Fluo-4. Activation of macrophages was achieved by focal puff administration of ATP. The effects of nicotine on activation of macrophages were evaluated and the nAChR involved was pharmacologically characterized. The proximity of cholinergic nerves to macrophages was quantified by confocal microscopy. Expression of β2 and α7 nAChR was evaluated by β2 immunohistochemistry and fluorophore-tagged α-bungarotoxin.

**Results:**

In 83% of macrophages cholinergic varicose nerve fibers were detected at distances <900nm. The ATP induced [Ca^2+^]_i_ increase was significantly inhibited in 65% or 55% of macrophages by 100µM or 10µM nicotine, respectively. This inhibitory effect was reversed by the β2 nAChR preferring antagonist dihydro-β-eryhtroidine but not by hexamethonium (non-selective nAChR-antagonist), mecamylamine (α3β4 nAChR-preferring antagonist), α-bungarotoxin or methyllycaconitine (both α7 nAChR-preferring antagonist). Macrophages in the stomach express β2 but not α7 nAChR at protein level, while those in the intestine express both receptor subunits.

**Conclusion:**

This study is the first *in* *situ* demonstration of an inhibition of macrophage activation by nicotine suggesting functional signaling between cholinergic neurons and macrophages in the stomach. The data suggest that the β2 subunit of the nAChR is critically involved in the nicotine-induced inhibition of these resident macrophages.

## Introduction

In 2000, Tracey and coworkers demonstrated that electrical stimulation of the vagus nerve provides a potent anti-inflammatory input to the spleen. In a mouse model of sepsis, vagal nerve stimulation (VNS) resulted in decreased pro-inflammatory cytokine production, an effect dependent on α7 nicotinic acetylcholine receptors (nAChRs) [1,2]. This so-called “cholinergic anti-inflammatory pathway” (CAIP) operates through adrenergic splenic nerves making synaptic-like contacts with β2 adrenergic receptor-expressing splenic T-cells. Subsequent release of acetylcholine (ACh) from these T-cells is responsible for the anti-inflammatory effect presumably by interacting with α7nAChR-expressing macrophages [1,2].

In 2005, we provided evidence that the CAIP also modulates the intestinal immune system. In a mouse model of postoperative ileus, we showed that VNS reduced intestinal manipulation-induced inflammation of the small intestine, a beneficial effect dependent on α7 nAChR but independent of the spleen [3,4]. These data suggest that the gut immune system is rather directly modulated by vagal nerve endings and/or enteric neurons. As resident intestinal macrophages are the main players triggering this inflammatory response [5], these cells represent the most likely target of the CAIP. In vitro studies using isolated peritoneal macrophages, peripheral blood mononuclear cell-derived macrophages or macrophage cell lines have indeed abundantly demonstrated that acetylcholine and nicotine reduce cytokine production [1-3,6,7] and increase phagocytosis [6]. Nevertheless, it remains questionable to what extent their phenotype really resembles that of the resident macrophages affected by enteric neurons, especially as receptor expression in macrophages may be up- or down-regulated as they have been isolated from their natural environment. Therefore, we decided to develop a technique that would allow us to study the effect of nicotine on the activation of resident macrophages in their natural environment. As macrophages activated by ATP, a well known danger signal for immune cells [8,9], reveal an increase in intracellular Ca^2+^, live Ca^2+^ imaging was chosen to study the resident macrophages in intact flat sheet mouse stomach preparations. This allowed us to compare ATP evoked Ca^2+^ transients ([Ca^2+^]_i_) in macrophages located in the smooth muscle layers before and after application of nicotine. We further used several antagonists with known preferences for specific nAChR subunits in order to provide further mechanistic insights into the roles of nicotine receptor expressing macrophages for the CAIP in the gut. These pharmacological findings were confirmed by immunohistochemistry. Finally, we analyzed the proportion of resident macrophages that are in close proximity of cholinergic nerve fibers.

## Methods

### Ethics Statement

All mouse work was conducted according to the German guidelines for animal care and welfare (Deutsches Tierschutzgesetz) and approved by the Bavarian state ethics committee (Regierung Oberbayern, which serves as the Institutional Care and Use Committee for the Technische Universität München) according to §4 and §11 Deutsches Tierschutzgesetz under the reference number 32-568-2.

### Tissue samples

Male 12-16 weeks old C57Bl/6 mice (Charles River, Sulzfeld, Germany) were killed by cervical dislocation. The stomach was harvested in ice-cold Krebs buffer containing (in mM) 117 NaCl, 4.7 KCl, 1.2 MgCl_2_ 6 H_2_O, 1.2 NaH_2_PO_4_, 25 NaHCO_3_, 2.5 CaCl_2_ 2 H_2_O and 11 glucose and adjusted to pH 7.4. The stomach was opened along the greater curvature and washed with ice-cold Krebs. Under microscopic inspection, the stomach was pinned down on a sylgard dish and the mucosa and submucosa were carefully removed. During the dissection, the tissue was continuously perfused with ice-cold Krebs solution to ensure viability of the tissue. Only the anterior or posterior half of the stomach was pinned on a small sylgard ring with a central opening of 100 x 200 mm^2^.

### Calcium imaging

Flat sheet mouse stomach preparations were incubated in modified Krebs solution (117 NaCl, 4.7 KCl, 1.2 MgCl_2_ 6 H_2_O, 1.2 NaH_2_PO_4_, 20 NaHCO_3_, 2.5 CaCl_2_ 2 H_2_O and 11 glucose) containing 30 μM of the fluorescent calcium indicator Fluo 4-acetoxymethyl (AM) (Invitrogen) and 1.25 mM probenecid (Sigma-Aldrich, Schnelldorf, Germany) for 2 h at room temperature in the dark and gassed with carbogen (95% O_2_ - 5% CO_2_). The sylgard ring was mounted in the recording chamber with serosal side of the stomach facing the bottom of the chamber. The chamber was connected to the perfusion system to enable continuous perfusion with carbogen-gassed Krebs solution at room temperature. A washout period of 1.5 h was allowed before the start of the experiment. The tissue chamber was mounted on an inverted epifluorescence microscope (Zeiss Axio Observer A1, Carl Zeiss, Jena, Germany) equipped with a high speed monochrome camera (Zeiss AxioCam HSm) and software (Zeiss Axio Vision 4.8) for acquisition and analysis. Fluo-4 was excited using a blue light emitting diode (LED) Luxeon III (3W, 470nm dominant wavelength, Philips Lumiled, Phillips, Hambur, Germany) and Fluo-4 signals were detected with a filter cube F26-514 Bright Line FITC BP (excitation: HC475/35, dichroic: 499, emission: HC530/43, AHF Analysentechnik, Tübingen, Germany) using X20 objective (A-Plan, NA = 0.25, Zeiss). The system measured relative changes in fluorescence (Δ F/F) of Fluo-4 monitoring changes in intracellular calcium ([Ca^2+^]_i_). Ca^2+^ transients were recorded starting 3 s before local administration of ATP for a total of 14.5 s with a frame rate of 2 Hz and exposure time of 200 ms. 

We used ATP as a tool for macrophage activation since it is a danger signal released locally at the site of inflammation [8,9] and since ATP is known to induce cytokine secretion from macrophages via increase of [Ca^2+^]_i_ [10–12].

ATP (Sigma-Aldrich) and nicotine (Sigma-Aldrich) were locally applied by pressure ejection from two micropipettes with durations of 200 ms and 10 sec, respectively. The position of the micropipettes ensured that the ejected volumes covered identical tissue regions. The micropipettes were filled with 1 mM ATP and 100 μM or 10 μM nicotine dissolved in Krebs solution containing 1.25 mM probenecid. According to previously published calibration curves, we estimate that any substance applied by pressure ejection pulses will be diluted by approximately 1:10 once it reaches the tissue [13].

Local administration of ATP and nicotine allowed measurement of responses at multiple regions (typically 4-5) in the same tissue. The position of the regions in the tissue was documented using the coordinate system displayed on the mobile microscope stage. The effect of nicotine on ATP induced calcium transients were again studied in the same regions after addition of various nAChR antagonists. After recording the responses, macrophages were visualized by vital incubation of the tissue with allophycocyanin (APC)-labelled rat anti-mouse anti-F4/80 antibody (1:250, eBioscience, Frankfurt, Germany) for 1 h, at room temperature in the dark and gassed with carbogen. The tissue was washed for 15 minutes. The microscope stage was repositioned to find the regions where we recorded from. Images of labeled macrophages were acquired using red Z-LED P4 (3.5 W) excitation source (625 nm dominant wavelength, Seoul Semiconductor) and filter cube F46-006 ET filter set (excitation: ET 620/60, dichroic: 660, emission: ET700/75, AHF Analysentechnik, Tübingen, Germany). The overlay of Fluo-4 signals and images of F4/80 positive macrophage allowed us to analyze the responses in individual macrophages.

### Immunohistochemistry

To label tissue resident macrophages, we first used the vital labeling protocol described above. The tissue was then fixed overnight at room temperature in a solution containing 4% formaldehyde and 0.2% picric acid in 0.1 M phosphate buffer, washed 3 X 10 minutes in phosphate buffer and finally incubated for 1 h in a solution containing 0.5% Triton X-100, 0.1% NaN_3_, 4% horse serum dissolved in PBS (all from Sigma-Aldrich). To label cholinergic varicosities, the tissue was incubated overnight in the blocking solution containing goat anti-vesicular acetylcholine transporter (VAChT; 1:1000; Merck-Millipore, Darmstadt, Germany). The tissues were washed 3 X 10 min in PBS and incubated for 1.5 - 2 h in the blocking solution containing Cy3-labeled anti-goat antibody (1:500; Dianova, Hamburg, Germany). The tissues were washed 3 X 10 minutes in PBS and mounted in anti-fade substance (20% PBS/NaN_3_, 80% glycerol) on poly-L-lysine-coated slides and coverslipped for viewing.

To label β2 nAChR in tissue resident macrophages, mucosa-free gastric whole mount preparations from wild-type and β2 nAChR knock-out [14] mice were fixed for 10 min in ice-cold PBS solution containing 4% paraformaldehyde (PFA). The tissues were then washed 2 X 10 min in PBS and incubated for 2 h in PBS containing 1% protease-free Albumin Bovine Fraction V albumin (BSA; Serva, Heidelberg, Germany) and 10% normal donkey serum (NDS; Jackson ImmunoResearch, Pennsylvania, USA). The tissues were incubated for 36 h with PBS containing 1% BSA, 5% NDS, rat anti-mouse F4/80 (1:500; clone BM8, BioLegend, San Diego, USA) and rabbit anti-mouse β2 nAChR (1:200; Santa Cruz Biotechnology, Heidelberg, Germany). The tissues were washed 3 X 10 min in PBS and incubated for 1 h in PBS containing 1% BSA, 5% NDS, Alexa Fluor® 647-labeled goat anti-rat antibody (1:1,000; Jackson ImmunoResearch, Pennsylvania, USA) and Cy3-labeled donkey anti-rabbit antibody (1:1,000; Chemicon, Millipore, Billerica, USA). The tissues were washed 3 X 10 minutes in PBS and mounted in slow-fade reagent (Invitrogen, Life Technologies, Gent, Belgium) on poly-L-lysine-coated slides and coverslipped for viewing.To label α7nAChR in tissue resident macrophages, a piece of mouse stomach and ileum were subjected to vital labeling using Cy5-labeled α-bungarotoxin (Invitrogen) at 0.1 μg/ml in RPMI 1640 medium (Lonza, Basel, Switzerland) at 4°C for 15 min [4]. The tissues were then fixed in PBS containing 4% PFA. The mucosa and submucosa were removed and the tissues were incubated for 2 h in blocking solution containing 1% BSA. The tissues were then incubated for 60 h in blocking solution containing rat anti-mouse F4/80 (clone BM8, BioLegend), followed by 3 X 10 min washes in PBS and incubated for 1 h in PBS containing 1% BSA and Cy3-labeled anti-rat antibody (1:1,000; Jackson ImmunoResearch, Pennsylvania, USA). Finally, the tissues were washed 3 X 10 minutes in PBS and mounted in slow-fade substance on poly-L-lysine-coated slides and coverslipped for viewing.

### Confocal microscopy and image analysis

Images were acquired using an LSM 510 (Carl Zeiss) confocal microscope with Plan-Neofluar x40/1.3 Oil DIC and Plan Apochromat x63/1.4 Oil DIC objectives. Laser wavelengths of 543 nm and 633 nm were used for the excitation of the fluorophores Cy3 and APC or Cy5, respectively. Cy3 and APC or Cy5 signals were detected using the filter sets BP 565-615 IR and BP 650-710 IR, respectively. 

Image stacks for the quantitative analysis using the x63 objective were scanned with an XY resolution of 1024×1024 that covered an area of 95.5×95.5 μm^2^. The first and last optical slices were taken at the top and bottom of the outer surface of a macrophage. The optical depth of each slice was 900 nm. Two consecutive slices overlapped for 500 nm. Usually, between 9 and 16 slices were generated resulting in a scan depth of 3.2-6.0 μm containing between 1-3 macrophages and VAChT positive fibers crossing macrophages. Image stacks were analyzed using Image J Pro.

Images of β2 and α7 nAChR-labeled macrophages were taken using the x63 objective and scanned with an XY resolution of 1024×1024 that covered an area of 95.5×95.5 μm^2^. The optical depth of the images was 900 nm.

### Pharmacology

To block action potential propagation in neurons, tetrodotoxin (Biotrend, Köln, Germany) was added to the perfusing Krebs solution at 1 µM. For pharmacological characterization, the following nicotinic blockers were added to the Krebs solution perfusing the tissue: 200 μM hexamethonium (Sigma-Aldrich), 100 μM mecamylamine (Sigma-Aldrich), 10 μM dihydro-β-erythroidine (DHBE; Sigma-Aldrich), 100 nM, 3 µM and 10 µM α-bungarotoxin (ABGT; Tocris) and 10 nM and 100 nM methyllycaconitine (MLA, Tocris). Hexamethonium, mecamylamine and DHBE were tested in the concentrations that were able to abolish nicotinic fast excitatory postsynaptic potentials in guinea pig myenteric neurons [15].The use of 10 nM and 100 nM MLA is based on concentration respectively used to block α7 subunit containing nAChR [16] and used to inhibit IL-6 secretion from isolated peritoneal macrophages [3].

### Data analysis and statistics

The maximal relative changes in fluorescence (Δ F/F) in response to ATP administration was expressed as % increase above basal fluorescence before ATP administration. The statistical analyses were performed with Sigma Plot 9.0 (Systat Software Inc, Erkrath, Germany). Data are presented as whisker plots with the median and the 25^th^ and 75^th^ percentiles as well as the 10^th^ and 90^th^ percentiles. Not normally distributed paired data were analyzed by the Wilcoxon signed rank test. Differences were considered statistically significant at *P* < 0.05. *N* numbers given in parentheses indicate numbers of macrophages/tissues studied, i.e. a result based on recordings of 20 macrophages in 5 tissues (equal to 5 animals) is presented as (20/5).

## Results

### Spatial relationship between tissue resident macrophages and cholinergic varicosities in mouse stomach

**Figure 1:**
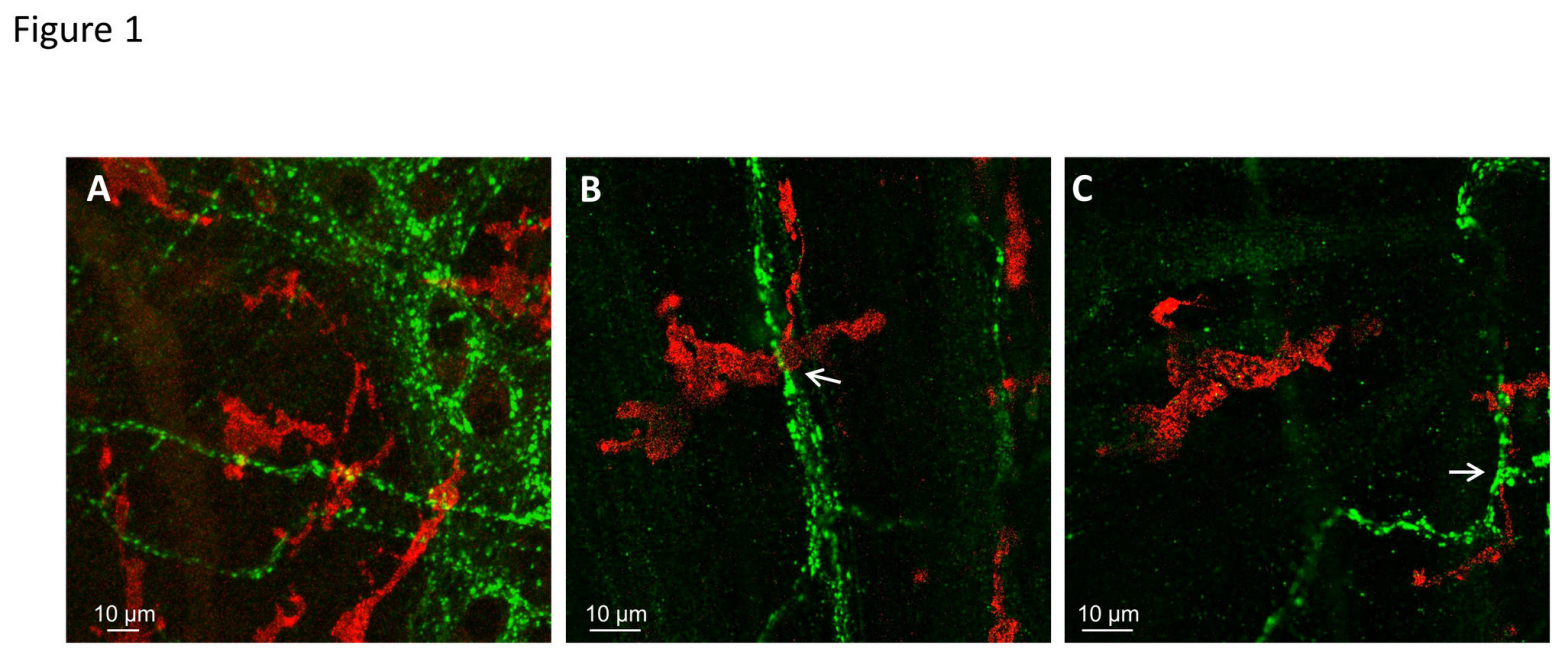
Confocal analysis of the proximity of macrophages to cholinergic varicosities in mouse stomach myenteric plexus-circular muscle layer region. F4/80 positive macrophages are labeled in red, VAChT positive acetylcholine-releasing sites are labeled in green. *A*, Two-dimensional maximal intensity projection of 6 confocal images (1.1 μm each, interval of 0.5 μm) showing close apposition of macrophages and cholinergic varicosities. *B* and *C*, Sections #-2 (B) and #-6 (C) show representative images of a confocal stack taken to evaluate proximity of macrophages to VAChT-positive nerve fibers. The two sections display two macrophages, each at less than 0.9 μm from VAChT-positive nerve fibers (marked by arrows).

We used confocal microscopy to assess the vicinity between F4/80-positive macrophages and VAChT-positive varicose cholinergic nerve fibers in the mouse stomach (Figure 1A). Detailed analysis revealed that 83% of the 41 macrophages studied are located within 900 nm to at least one varicose cholinergic nerve fiber (Figure 1B-C).

### Reproducibility of ATP evoked [Ca^2+^]_i_ signals in tissue resident macrophages

**Figure 2:**
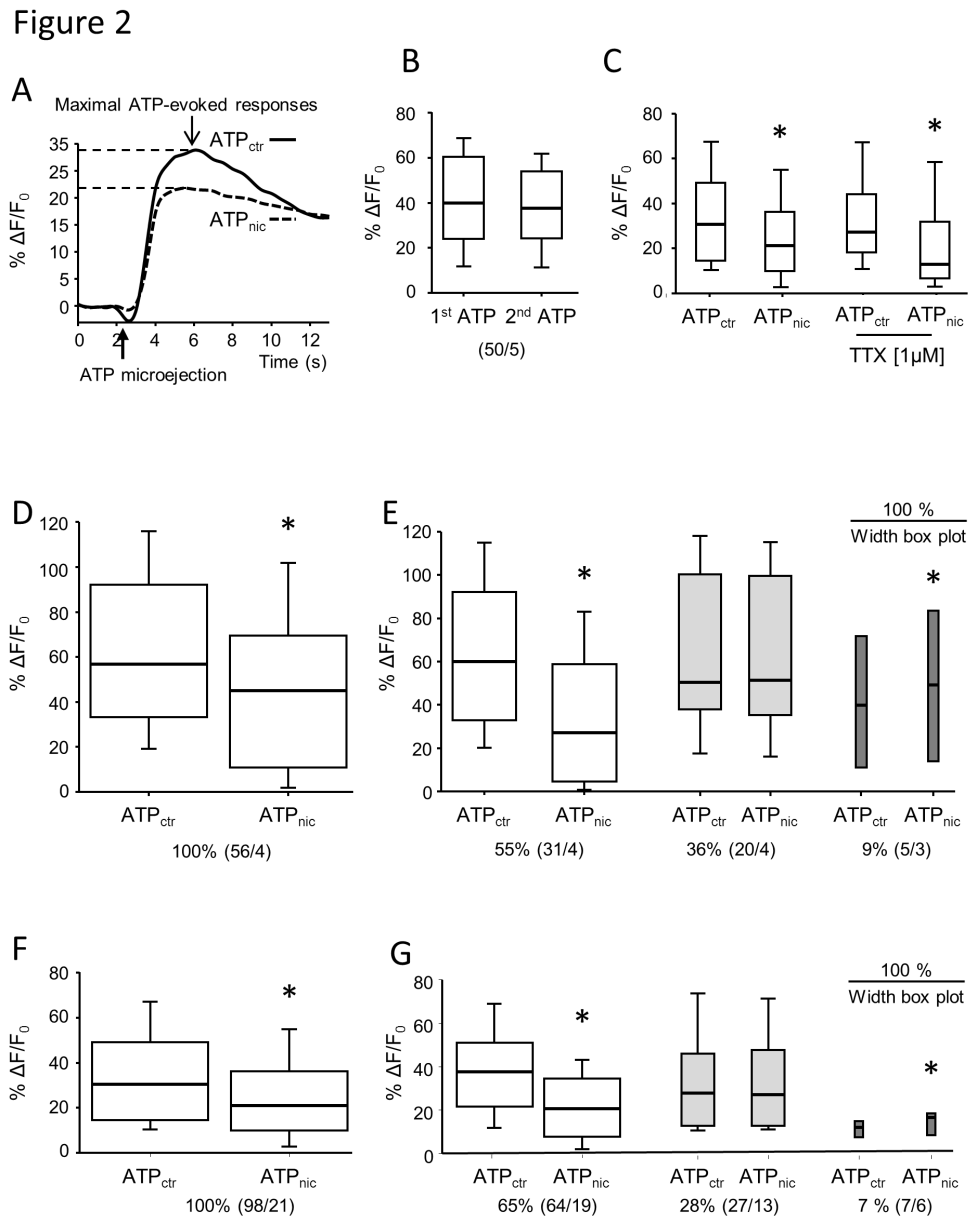
Reproducibility of ATP-evoked macrophage activation and effect of nicotine administration on macrophage activation. *A*, The trace depicts a representative [Ca^2+^]_i_ signal in a single macrophage in response to local microejection of ATP for 200 ms. Maximal increase in relative changes in Fluo-4AM fluorescence (%Δ*F*/*F*) was used for further analysis. *B*, ATP induced [Ca^2+^]_i_ responses are reproducible as there was no difference between amplitudes of [Ca^2+^]_i_ signals evoked by two consecutive ATP applications (10 minutes apart, ATP_1st_ and ATP_2nd_) (Wilcoxon Signed Rank test, *P* > 0.05). *C*, The nicotine inhibition remained in the presence of the nerve blocker tetrodotoxin (TTX, Wilcoxon Signed Rank test, * *P* = 0.002) suggesting that the attenuation of the ATP induced [Ca^2+^]_i_ signal did not involve nicotinic synapses in the myenteric plexus. *D*-*G*, Nicotine (10 μM in *D* and *E*; 100 μM in *F* and *G*) significantly inhibited the ATP-evoked [Ca^2+^]_i_ signal in macrophages (Wilcoxon Signed Rank test, * *P* < 0.001). Based on the nicotine effects on ATP-evoked [Ca^2+^]_i_ signals macrophages were subdivided in three groups. *D* and *F* show the significant attenuation induced by 10 µM and 100 µM nicotine, respectively, when the signals from all macrophages were analyzed. *E* and *G*, with both concentrations of nicotine the majority of macrophages were inhibited (white boxes), in some we did not observe any change in signal (light grey boxes) and in a minority the [Ca^2+^]_i_ signal was increased (dark grey boxes). The width of the box plots is proportional to the size of the subpopulations. ATP_ctr_ labels the maximum [Ca^2+^]_i_ amplitude to a control ATP administration. ATP_nic_ labels the maximum [Ca^2+^]_i_ amplitude to ATP application immediately after a 10 sec nicotine administration 10 minutes after the control ATP application. Numbers in parenthesis indicate number of macrophages / number of preparations (equal to number of animals). * *P* < 0.001 Wilcoxon Signed Rank test.

Microejection of ATP induced a strong, fast onset [Ca^2+^]_i_ transient in macrophages that reached its peak 8-10 sec after the application followed by a slow return to baseline [Ca^2+^]_i_ levels (Figure 2A and Movie S1). Since there was not always full recovery to baseline levels during the recording period, the maximal [Ca^2+^]_i_ signal was used for the analysis of all experiments. No tachyphylaxis was observed since the maximal amplitude of [Ca^2+^]_i_ signal in response to a second ATP administration, 10 minutes after the first one, did not differ from the initial maximal response (Figure 2B).

### Effect of nicotine on [Ca^2+^]_i_ signals in tissue resident macrophages

To study the effect of nicotine on ATP evoked [Ca^2+^]_i_ signals we microejected nicotine for 10 sec and immediately re-applied ATP onto the same region. One rational to use nicotine as selective, non-degraded nAChR agonist was to mimic nicotinic receptor activation by release of acetylcholine from cholinergic neurons. Analyzing the changes in [Ca^2+^]_i_ in all macrophages revealed that nicotine significantly reduced the ATP evoked [Ca^2+^]_i_ signals (Figure 2D and F). A more detailed analysis of the effects of nicotine on ATP-induced [Ca^2+^]_i_ transients revealed three populations of macrophages (Figure 2D-G). Nicotine at 10 and 100 µM attenuated the ATP-evoked [Ca^2+^]_i_ signals in 55% and 65% of macrophages, respectively. The [Ca^2+^]_i_ signal remained unchanged in 36% and 28% of macrophages after application of 10 μM and 100 μM nicotine, respectively. In a small subset, 10 μM and 100 μM nicotine potentiated the ATP-evoked [Ca^2+^]_i_ response (9% and 7% of macrophages, respectively).

To avoid any bias further analysis is based on nicotine effects on all macrophages independent of whether the ATP induced [Ca^2+^]_i_ signal was decreased, increased or unchanged.

### Role of neurons in the nicotinic evoked attenuation of macrophage activation

Nicotine directly activates enteric neurons and we addressed the possibility that activation of close-by myenteric neurons contributed to the attenuated [Ca^2+^]_i_ responses in macrophages. We did not find any evidence for such an indirect inhibitory pathway because the attenuation of the ATP-evoked [Ca^2+^]_i_ signal by nicotine remained in the presence of tetrodotoxin (Figure 2C). It is noteworthy though that the proportion of those macrophages where nicotine did not alter ATP evoked [Ca^2+^]_i_ signals were dramatically reduced (28% versus 6%). At the same time, macrophages in which nicotine inhibited or potentiated ATP evoked [Ca^2+^]_i_ signals increased from 65% to 71% and from 7% to 23%, respectively.

### Pharmacological characterization of the inhibitory effect of nicotine on tissue resident macrophages

**Figure 3:**
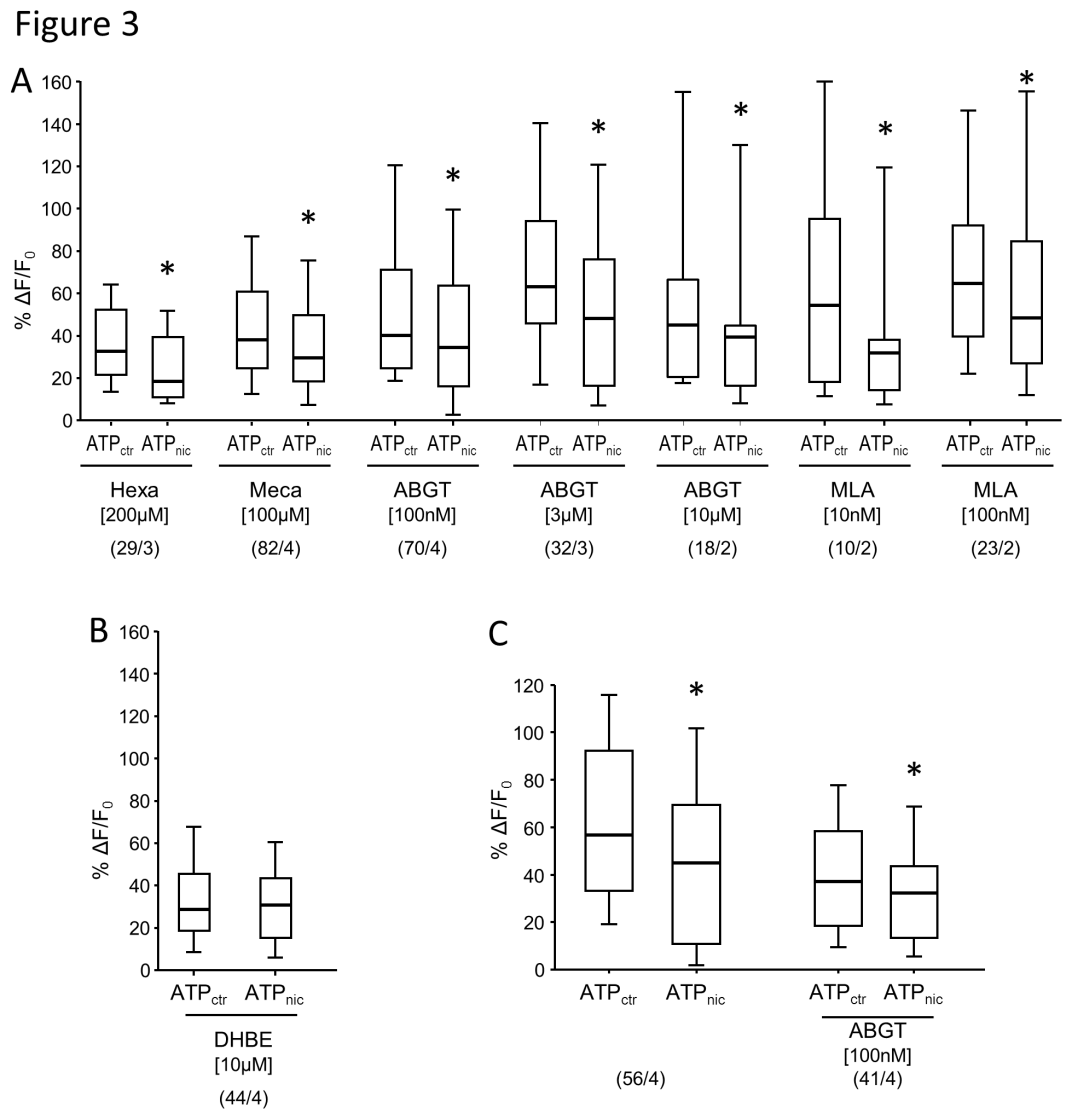
Pharmacology of the inhibitory effect of nicotine on ATP-evoked macrophage activation. *A*, In presence of hexamethonium (Hexa), mecamylamine (Meca), different concentrations of α-bungarotoxin (ABGT) or different concentrations of methyllycaconitine (MLA), 100 µM nicotine still had a significant inhibitory effect on ATP-evoked [Ca^2+^]_i_ signals in macrophages (Wilcoxon Signed Rank test, * *P* < 0.001). *B*, In contrast, DHBE reversed the inhibitory effect of nicotine (Wilcoxon Signed Rank test, *P* = 0.156). *C*, ABGT did not reverse the inhibitory effect of 10 µM nicotine on ATP-evoked macrophage activation (Wilcoxon Signed Rank test, * *P* < 0.001). Please note that the scatter plots illustrating the inhibitory effect of 10 µM nicotine on ATP responses are identical to those in Figure 2 D. We show them here again as we used the same preparations, but different regions, to test the effect of 100 nM ABGT. For all panels: ATP_ctr_ labels the maximum [Ca^2+^]_i_ amplitude to a control ATP administration. ATP_nic_ labels the maximum [Ca^2+^]_i_ amplitude to ATP application immediately after a 10 sec nicotine administration 10 minutes after the control ATP application. Numbers in parenthesis indicate number of macrophages / number of preparations (equal to number of animals).

The activation of macrophages by ATP and the inhibition of the ATP response by 100 µM nicotine was reliably recorded in each of the 21 preparations illustrated in Figure 2F. This allowed us to perform antagonists studies without the need to restudy the inhibitory response in those macrophages treated with the antagonists. Moreover, we thereby reduced the number of recording periods to a level that did not compromise signal strength and guaranteed reproducible ATP responses. In order to study the nAChR-subunits involved in the inhibitory effect of nicotine, we tested five different blockers with known subunit preferences [17] (Figure 3). The inhibitory effect of nicotine on ATP-evoked [Ca^2+^]_i_ responses was unchanged in the presence of the non-selective ganglionic nAChR antagonist hexamethonium, the α3β4 nAChR-preferring antagonist mecamylamine or α7 nAChR-preferring antagonists α-bungarotoxin and methyllycaconitine (Figure 3A). However, the β2 nAChR-preferring antagonist di-hydro-β-eryhtroidine reversed the inhibitory effect of nicotine on ATP-evoked [Ca^2+^]_i_ responses in macrophages (Figure 3B). 

Although we used ABGT at concentrations that have been described to reliably block α7 nAChR in neurons and isolated alveolar macrophages [18], we were concerned that it may be unable to antagonize the inhibitory effect of nicotine due to unfavorable competition at the binding site. However, this seems unlikely because even at concentrations of 3 µM and 10 µM ABGT was not able to reverse the inhibitory effect of nicotine on ATP evoked [Ca^2+^]_i_ responses (Figure 3A). ABGT did also not reverse the attenuation evoked by 10 µM nicotine (Figure 3C). 

### Labeling of β2 but not α7 nAChR in tissue resident macrophages

**Figure 4:**
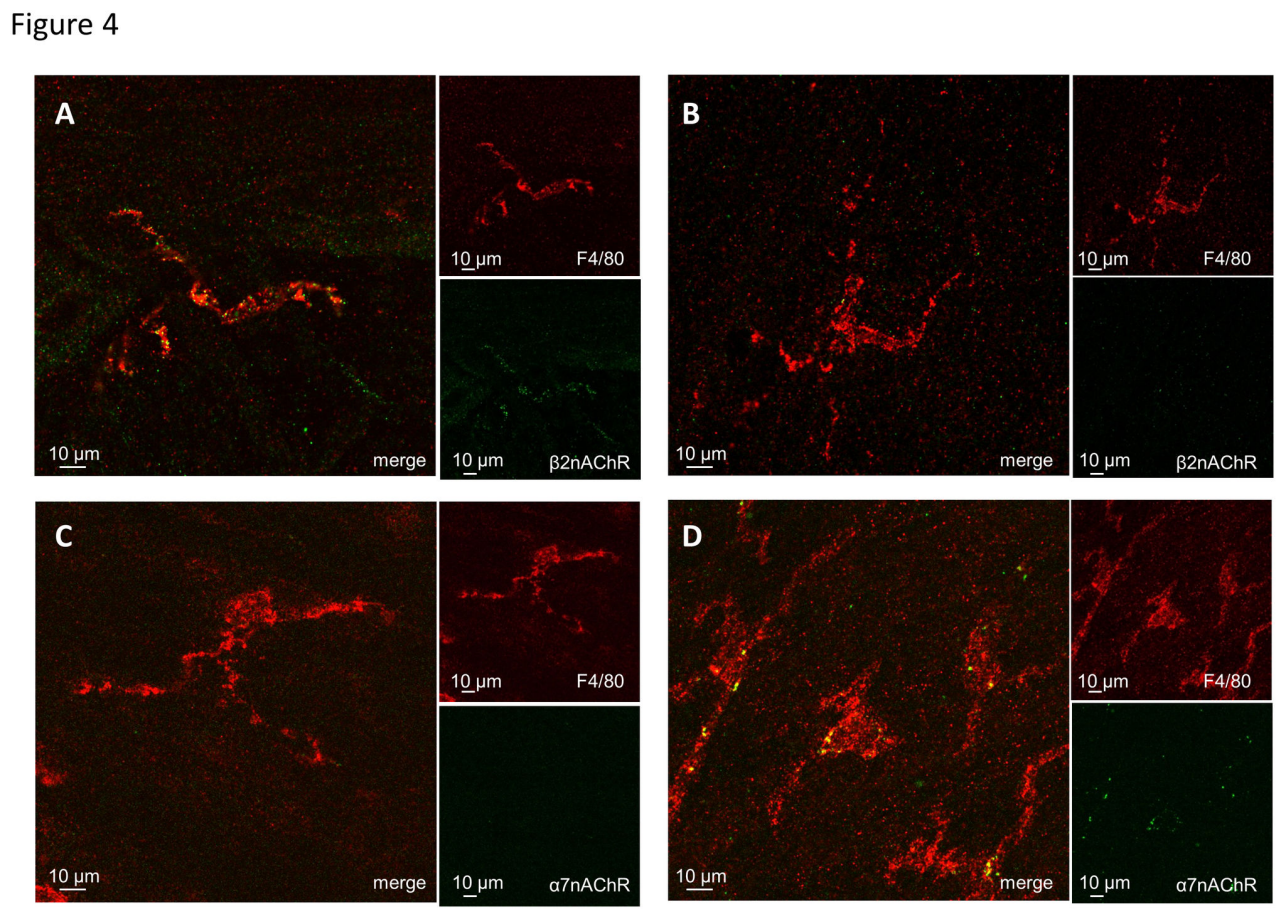
Labeling of β2 and α7 nAChR subunits in tissue resident macrophages in the stomach and ileum. Tissue resident macrophages were immunohistochemically labeled for F4/80 (red). A; Resident macrophages in stomach muscularis of a wild-type mouse were β2 nAChR-immunoreactive (green). B; Lack of β2 nAChR-immunoreactivity (green) in resident macrophages of β2 nAChR knock-out mouse confirmed the specificity of the anti-mouse β2 nAChR antibody. C; Vital labeling with Cy5-conjugated α-bungarotoxin (ABGT; false color coded in green) revealed no α7 nAChR expression in resident macrophages in mouse stomach muscularis. D; In the mouse ileum ABGT labeled (false color coded in green) muscularis macrophages. This staining was used as a positive control for the α-bungarotoxin vital labeling protocol.

To provide additional evidence for the involvement of β2 nAChR but not of α7 nAChR in the inhibitory effect of nicotine on ATP-evoked [Ca^2+^]_i_ responses, immunohistochemical labeling of β2 nAChR and vital labeling of α7 nAChR by ABGT in resident macrophages of stomach muscularis were performed (Figure 4). The majority of resident macrophages of stomach muscularis were β2 nAChR-immunoreactive (Figure 4A) supporting the observed antagonistic effect of DHBE on nicotinic inhibition of ATP responses. The used antibody for β2 nAChR is specific because of the absence of β2 nAChR immunoreactivity in a β2 nAChR knockout mouse (Figure 4B).

Vital labeling of α7 nAChR by ABGT revealed absence of α7 nAChR in tissue resident macrophages in the mouse stomach (Figure 4C), In contrast, intestinal macrophages were labeled by ABGT (Figure 4D). The lack of α7 nAChR in stomach muscularis resident macrophages confirmed the absence of antagonism of α7 nAChR-preferring blockers ABGT and MLA on the inhibitory effect of nicotine on ATP evoked [Ca^2+^]_i_ responses in these cells.

## Discussion

To date, the effect of nicotine has been investigated only in isolated macrophages or macrophage cell lines. Here we have developed an *in vitro* model of mouse stomach muscle-myenteric plexus preparation to study the effect of nicotine on tissue resident macrophages within their natural environment. This present study is therefore the first to show that nicotine directly inhibits activation of tissue resident macrophages through β2 subunit containing nAChR and thereby provides the basis for functional signaling between cholinergic neurons and macrophages in the gut. Our conclusion is supported by several lines of evidences. Firstly, ATP evoked [Ca^2+^]_i_ transients in macrophages is significantly reduced by nicotine even in the presence of the nerve blocker tetrodotoxin. Secondly, the nicotine-induced attenuation of ATP responses in macrophages was reversed by DHBE, but not by hexamethonium, mecamylamine, ABGT or MLA. The pharmacological findings were supported by the demonstration of β2-positiv but ABGT-negative nAChR subunits on gastric macrophages. Thirdly, the majority of macrophages were in direct vicinity of varicose cholinergic nerve fibers. Similar close proximity of macrophages to nerve fibers was observed in the rat intestinal muscularis [3].

Our criterion used to define close proximity between macrophages and cholinergic nerve fibers (900 nm distance) agrees with the concept of extrasynaptic communication. According to this concept a diffusion based volume transmission may occur at 100 nm to µm distances between the source and the target [19]. We assume that most, if not all, cholinergic nerves in close vicinity to macrophages originated from myenteric neuronal cell bodies based on our previous observation that vagal fibers do not contact intestinal resident macrophages [3], This is also supported by the findings that gastric vagal efferent fibers almost exclusively terminate in enteric ganglia [20], where they activate the majority of myenteric neurons through nicotinic receptors [15]. 

The CAIP represents a physiological system to control macrophage activation during inflammatory conditions. The anti-inflammatory effect of CAIP activation has been shown *in vivo* by vagal nerve stimulation in mouse models of sepsis and postoperative ileus [2,3] and *in vitro* by nicotine administration to isolated, lipopolysaccharide -stimulated macrophages [1-4,21]. In the present study, we found that nicotine reduced the ATP-induced increase in intracellular Ca^2+^ in resident macrophages in the stomach. Interestingly, this effect was reversed by the β2 nAChR subunit preferring antagonist DHBE suggesting the involvement of this subunit in the nicotine-mediated modulation of the resident macrophages. This observation is consistent with our previous finding that DHBE reversed nicotine-induced inhibition of tumor necrosis factor-α (TNF-α) release and increased phagocytosis in isolated peritoneal macrophages [6]. In line, cytokine production of human neuroblastoma cell line stably transfected with α4β2 nAChR is significantly reduced by nicotine pretreatment [22]. Although these data suggest that α4β2 nAChRs may mediate the effect of nicotine on the ATP-induced increase in intracellular Ca^2+^, recent evidence indicates that β2 subunits can also assemble with other α subunits, including α7 subunits. A recent publication discussing the electrophysiological properties of α7β2 nAChR expressed in human epithelial cell lines, showed that low concentrations of DHBE antagonized α7β2 nAChR but not α7 nAChR [16]. These and other data on the pharmacological profile of DHBE would suggest that DHBE is a highly selective antagonist of β2 nAChR but does not reliably discriminate between various assemblies of α3, α4 or α7 with β2. However, our immunohistochemical and vital labeling of gastric resident macrophages would not favor involvement of α7 nAChR because gastric macrophages were not labeled by ABGT but expressed the β2 nAChR subunit. 

Nevertheless, we opt for a rather conservative interpretation of our data and conclude that β2 nAChR are critically involved in nicotine inhibition of macrophage activity although it is likely that at the concentration used in our study DHBE preferably blocks α4β2 nAChR. Our preparation is ideally suited to address in future studies the possible contribution of α7β2 nAChR by investigating the effect of nicotine in tissue resident macrophages from α7 nAChR, β2 nAChR and α7β2 nAChR double knock-out mice. Similar strategies should be used to study the significance of an α4β2 nAChR.

It is important to note that the antagonistic action of DHBE is not selective for macrophages as DHBE, like hexamethonium and mecamylamine, also blocks nicotinic synapses in gastric myenteric neurons [15]. Nevertheless, the nicotinic receptors on macrophages must possess different properties than those in enteric neurons since neither hexamethonium nor mecamylamine reversed the nicotine induced attenuation of ATP-evoked responses in macrophages. Hexamethonium and mecamylamine exert their actions by clogging the pore of the nicotinic channel [23–25]. Their inability to reverse the nicotine inhibition of macrophages may suggest the existence of an atypical nAChR in macrophages. Indeed, recording of Ca^2+^ transients in response to nicotine administration revealed increased [Ca^2+^]_i_ in only 13% of macrophages (6 out of 45 macrophages; data not shown). This population is much smaller that the proportion of macrophages in which nicotine modulated the ATP evoked [Ca^2+^]_i_. 

Abundant evidence suggests that α7 nAChR plays a crucial role in the nicotine-induced reduction in macrophage cytokine production [2,3,6,26]. Previously, we indeed demonstrated that nicotine failed to reduce TNF-α production in peritoneal macrophages of α7 nAChR knockout mice [6], whereas the anti-inflammatory effect in the small intestine of vagus nerve stimulation in our model of postoperative ileus is lost in these KO mice [4]. In the present study, however, the effect of nicotine on ATP-induced macrophage activation was not blocked by the α7 nAChR preferring blockers ABGT and MLA, arguing against the involvement of α7 nAChRs. This is further supported by the lack of labeling of stomach muscularis macrophages by α7 nAChR-preferring α-bungarotoxin. In the intestine, however, we did observe α-bungarotoxin positive labeled muscularis macrophages [4 and this study], indicating region-specific differences in the phenotype of these immune cells.

Although the apparent lack of ABGT-sensitive α7 nAChR in our study appears contradictory to our previous findings, we are quite confident that these data are not due to the inability of ABGT to compete for the binding site as the same concentration used in our study blocks neuronal α7 nAChR in the brain and in isolated alveolar macrophages [18]. Even a 100 times higher concentration ABGT did not prevent the nicotine-induced inhibition of macrophage activation. One possible explanation could be that the expression of α7 nAChRs in the stomach differs from that in the small intestine. Alternatively, although speculative, we like to put forward the hypothesis that the contribution of α7nAChR may be dependent on the activation level of macrophages. It is striking that involvement of α7 nAChR has been described under inflammatory conditions or after isolation procedures which very likely stress macrophages causing a switch in their phenotype. For example, chronic neuroinflammation or lipopolysaccharide caused an increased expression of α7 nAChR in macrophages [27,28]. Our preparations required rather mild dissection procedures which may not sufficiently challenge macrophages to express α7 nAChR.

In conclusion, we have established a mouse stomach preparation to study cholinergic modulation of macrophage activity. Nicotine inhibited ATP-evoked [Ca^2+^]_i_ increase in tissue resident macrophages, an effect which was abolished by the β2-subunit preferring nAChR antagonist DHBE but not by hexamethonium, mecamylamine or the α7-subunit preferring nAChR antagonists ABGT or MLA.

## Supporting Information

Movie S1 **This movie shows changes in [Ca^2+^]_i_ after microejection of ATP in flat sheet mouse stomach preparations containing the muscle layers and the myenteric plexus.** Green flashes indicate increase in Fluo-4AM fluorescence as a result of an increase in [Ca^2+^]_i_. The macrophages in red were visualized by vital labeling with APC-conjugated anti-F4/80 antibody and overlayed with the Fluo-4AM signal. ATP evoked increase in [Ca^2+^]_i_ is clearly visible in two macrophages in the field of view. The movie shows a speeded-up sequence of 26 images (sampling frequency 2Hz, original length was 14 sec).
(WMV)
